# 2-Allylaminothiazole and 2-allylaminodihydrothiazole derivatives: synthesis, characterization, and evaluation of bioactivity

**DOI:** 10.1007/s00706-015-1539-z

**Published:** 2015-08-05

**Authors:** Renata Studzińska, Aleksandra Karczmarska-Wódzka, Anna Kozakiewicz, Renata Kołodziejska, Renata Paprocka, Marcin Wróblewski, Beata Augustyńska, Bożena Modzelewska-Banachiewicz

**Affiliations:** Department of Organic Chemistry, Faculty of Pharmacy, Collegium Medicum in Bydgoszcz, Nicolaus Copernicus University in Toruń, Toruń, Poland; Department of Biochemistry, Faculty of Medicine, Collegium Medicum in Bydgoszcz, Nicolaus Copernicus University in Toruń, Toruń, Poland; Department of Crystallochemistry and Biocrystallography, Faculty of Chemistry, Nicolaus Copernicus University in Toruń, Toruń, Poland

**Keywords:** Heterocycles, Thiazole derivatives, Organic synthesis, Structure–activity relationships, X-ray structure determination

## Abstract

**Abstract:**

Some reactions of selected chlorooxoesters and haloesters with a 1-allylthiourea under various conditions have been performed. The reactions have been performed in methanol in alkaline and neutral environment. Condensation of 1-allylthiourea with chlorooxoesters has been further led via acetal as intermediate compound. As a result, the compounds containing thiazole and a 4,5-dihydrothiazole ring with a good yield have been obtained. The structures of the compounds were verified by ^1^H NMR, ^13^C NMR as well as X-ray diffraction analysis. Due to the potential biological activity of the synthesized compounds, the parameters of their bioavailability have been determined, and the probability of pharmacological action has been defined. All of the obtained compounds fulfilled the rule of five, which indicate their good absorption after oral intake. The probability of pharmacological action and potential targets calculated for the obtained compounds show that they can be potential drugs.

**Graphical abstract:**

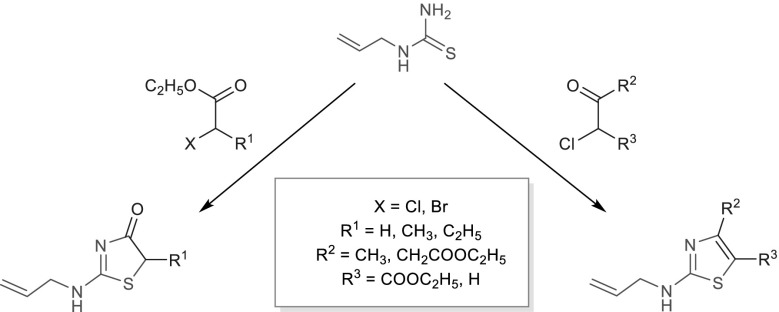

## Introduction

**Fig. 1:**
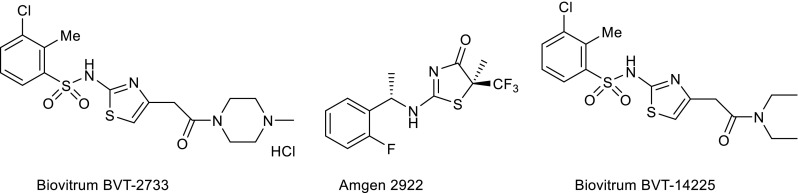
11β-Hydroxysteroid dehydrogenase type 1 inhibitors containing a thiazole and dihydrothiazole ring

The thiazole and 4,5-dihydrothiazole derivatives exhibit promising biological activity. The attention should be paid to, among others, biovitrum BVT-2733, biovitrum BVT-14225 [1–4], and amgen 2922 [4–6], which are described as inhibitors of 11β-hydroxysteroid dehydrogenase type 1 (Fig. 1). 11β-Hydroxysteroid dehydrogenase type 1 regulates glucocorticoid action, and the inhibition of this enzyme is a viable therapeutic strategy for the treatment of type 2 diabetes and the metabolic syndrome. Furthermore, compounds containing a thiazole ring are used as inhibitors of thymidylate synthase [7, 8]. Due to their biological activity, it is worth concentrating on the efficient method of the thiazole and dihydrothiazole ring formation.

One of the ways of forming a dihydrothiazole ring is the reaction of thiourea or its derivatives with haloacids. Such reactions have been described before [9, 10]. They have been carried out in various solvents, i.e., water, pyridine, benzene, and acetonitrile. The obtained products had the form of hydrogen halides. To obtain dihydrothiazoles, hydrogen halides were treated in alkaline conditions: sodium carbonate or aqueous solution of ammonia.

Due to the previously conducted condensation reactions of 1-allylthiourea (**1**) with oxoesters in alkaline conditions, which have allowed us to obtain the *N*-allylthiouracil derivatives [11, 12], we have become interested in carrying out the reactions of haloesters with **1** in similar conditions. Encouraged by high efficiency of the synthesized products as well as the easiness of their extracting, we have expanded the conducted synthesis applying chlorooxoesters.

## Results and discussion

### Chemistry

**Fig. 2:**
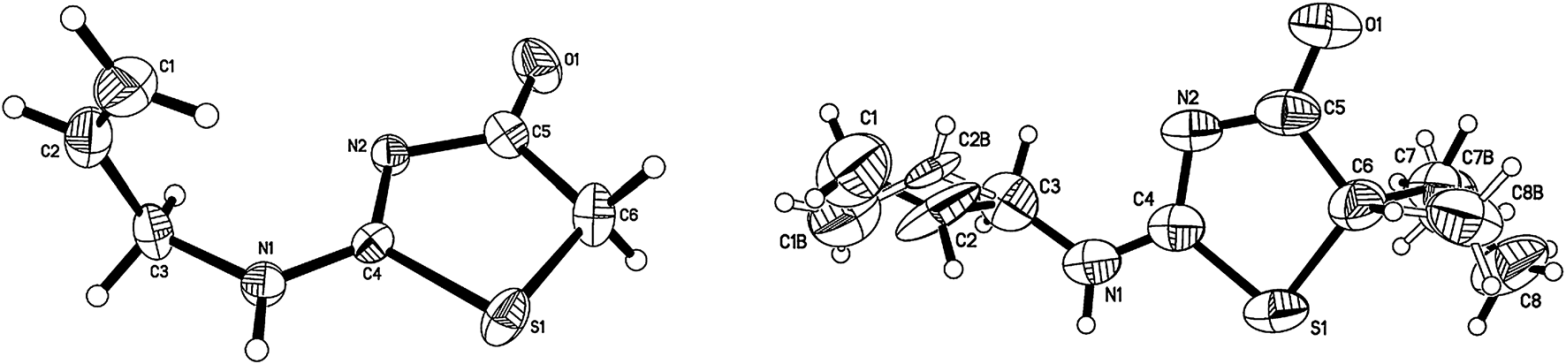
X-ray structure of **5** (*left*) and **7** (*right*)

The condensation reactions for three different haloesters **2**–**4** were carried out in alkaline conditions (Table 1). The reaction of **1** with haloesters was carried out in methanol with a 10 % excess of ester and a double excess of sodium methoxide (procedure A). The products were extracted in a yield of 50–65 %. Additionally, the analogous reactions without the base afforded products **6** and **7** in lower yields (40–47 %), whereas only trace amounts of product **5** were obtained from ester **2** (procedure B). The structures of **5**–**7** were verified by ^1^H and ^13^C NMR spectral data, as well as mass spectra. The structures of **5** and **7** were also verified by X-ray diffraction analysis (Fig. 2).

**Table 1:** The condensation reaction of **1** with 2-haloesters **2**-**4**

No.	X	R^1^	Procedure^a^	Time/h	Yield of **5**–**7**/%	M.p. of **5–7**/°C
**2**	Cl	H	A B	8 24	50 Trace	89–90^b^
**3**	Br	CH_3_	A B	1 1	68 47	75–76
**4**	Br	C_2_H_5_	A B	3 2	65 40	77–78

^a^Procedure A: MeOH, MeONa, the boiling point; procedure B: EtOH, the boiling point

^b^Ref. [9] 102–103 °C

**Fig. 3:**
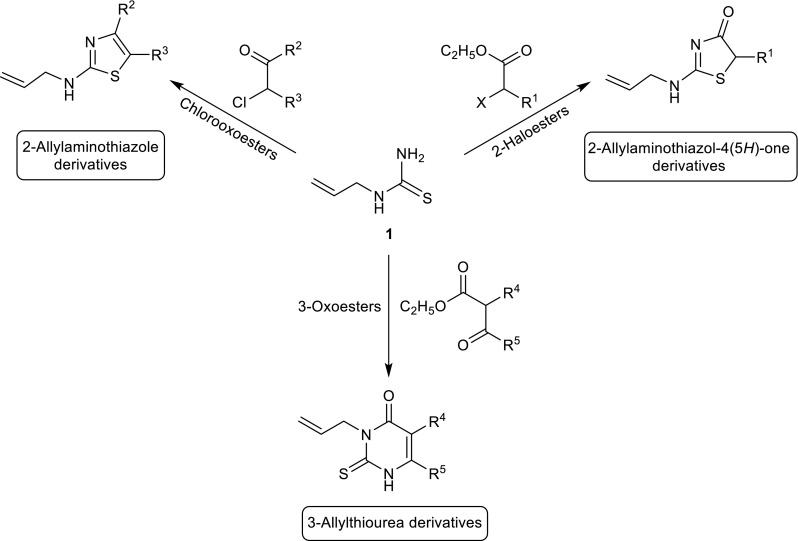
The reaction of **1** with 3-oxoesters, chlorooxoesters, and haloesters

The above-mentioned highly selective reactions in a good yield in alkaline conditions have encouraged us to carry out the analogous reactions with the use of **1** and chlorooxoesters. The condensation reactions of **1** with 3-oxoesters lead to the formation of 3-allyl-2-thiouracil (Fig. 3) [13, 14]. The presence of a chlorine atom in the oxoester molecules **9** and **10** causes a carbonyl group and a halogen atom to be involved in the reaction, while an ester group is not (Fig. 3; Table 2). Furthermore, the nucleophilic substitution reaction occurs on the sulfur atom, but does not occur on the nitrogen atom, as in the case of addition elimination reaction in the ester group of a 3-oxoester molecule. Consequently, a five (not six)-membered ring containing nitrogen and sulfur atom is formed, in the same way as while using haloesters **2**–**4** (Table 1). As a result of addition of **1** to the carbonyl group of chlorooxoester, and then the elimination of water, the cyclic products **11**, **12**, containing not one but two double bonds in the ring, were formed.

**Table 2:** The condensation reaction of **1** with chlorooxoesters

No.	R^2^	R^3^	Procedure^a^	Time/h	Yield of **11**–**12**/%	M.p. of **11**–**12**/ °C
**9**	CH_3_	COOC_2_H_5_	A B C	38 1 1	4 42 82	104–105^b^
**10**	CH_2_COOC_2_H_5_	H	A B C	13 1 1	15 35 60	42–43

^a^Procedure A: MeOH, MeONa, procedure B: EtOH, procedure C: step 1—MeOH, HC(OCH_3_)_3_, step 2—EtOH, H_2_SO_4_

^b^Ref. [15] 70–73 °C, Ref. [16] 109–110 °C

The reaction of **1** with chlorooxoesters and haloesters was carried out at the same alkaline conditions (procedure A). In the first case the products were obtained with a much lower yield (4–15 %), which is probably due to two simultaneous condensation reactions: aldol and Claisen condensation reaction (Table 2).

**Fig. 4:**
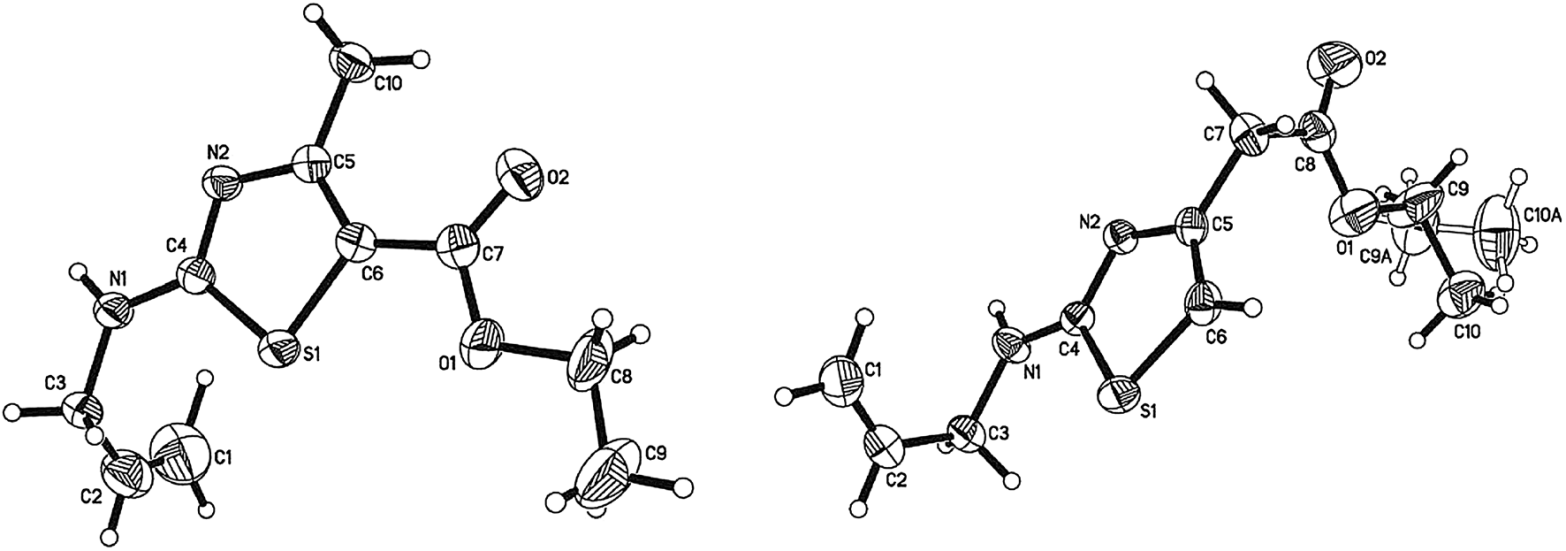
X-ray structure of **11** (*left*) and **12** (*right*)

The poor yield of products **11** and **12** made us change the conditions of conducting the reactions. The reaction of **1** with chlorooxoesters in ethanol let us obtain products **11** and **12** in higher yields (35–42 %) (procedure B). A further increase of a yield was obtained due to transforming a carbonyl group into acetal, followed by the reaction of obtained acetals from **1** in acidic environment, according to the procedure described above (procedure C) [14]. The structures of **11** and **12** were verified by ^1^H and ^13^C NMR spectral data, mass spectra, as well as X-ray diffraction analysis (Fig. 4).

### Evaluation of biological activity

Due to the biological activity of the compounds containing thiazole and dihydrothiazole rings [1–8], we have calculated the parameters conditioning the bioavailability of the synthesized compounds and their probable activity.

Lipinski’s rule of five describes molecular properties important for drug pharmacokinetics in the human body, especially their oral absorption. The rule says, that an orally active drug must not violate more than one of the following criteria: ≤5 hydrogen donors (nOHNH), ≤10 hydrogen acceptors (nON), MW ≤ 500 Da, log P_calc_ ≤5 [17]. Those properties calculated with Molinspiration program [18] are presented in the Table 3. All compounds **2**–**4**, **11**, **12** fulfilled the rule of five, which indicate their good absorption after oral intake.

**Table 3:** The value of molecular weight, miLog*P*, hydrogen donors and acceptors, and TPSA calculated by Molinspiration program

	**5**	**6**	**7**	**11**	**12**
Molecular weight	156.21	170.24	184.26	226.30	226.30
miLog*P*	0.361	0.724	1.226	1.527	2.150
nOHNH	1	1	1	2	1
nON	3	3	3	4	4
TPSA/Å^2^	41.46	41.46	41.46	62.23	51.22

Topological polar surface area (TPSA) becomes another popular indicator of drug absorbance [19]. TPSA values lower than 140 Å^2^ suggest good cell membrane permeability of derivatives **2**-**4** and **11**, **12**. Moreover, calculated TPSA values below 60 Å^2^ suggest high chances to penetrate blood–brain barrier for compounds **2**–**4** and **12**.

The probability of pharmacological action and potential targets for compounds **2**–**4** and **11**, **12** were calculated by PASS online program [20], which is based on analysis of structure–activity relationships (SAR) of chemical compounds and predicts over 4000 kinds of biological activities, including pharmacological effects, mechanisms of action, toxic and adverse effects with average accuracy above 95 % [21]. The selected results of the predicted activities and mechanisms of action of **2**–**4** and **11**, **12** are presented in Table 4.

**Table 4:** Probabilities of biological activity and action mechanisms for compounds **5**–**7**, **11**, **12**

		Pa/%				
		**5**	**6**	**7**	**11**	**12**
Pharmacological action	Antieczematic	36.6	40.2	55.6	68.5	89.6
	Mucomembranous protector	78.5	55.7	61.6	62.5	83.2
	Antiulcerative	55.6	53.7	29.8	65.9	75.2
	Antiviral	49.9	60.1	57.2	52.9	66.3
	Radioprotector	46.1	65.7	67.4	np	21.4
Mechanism of action	Chloride peroxidase inhibitor	71.0	61.8	52.0	43.9	np
	Muramoyltetrapeptide carboxypeptidase inhibitor	35.0	30.4	30.9	49.8	75.5
	Membrane permeability inhibitor	67.1	55.2	45.5	68.9	46.6
	Cl^−^-transporting ATPase inhibitor	67.7	59.4	54.3	43.1	36.2
	Gastrin inhibitor	66.6	69.3	60.7	55.9	51.5

*np* Not predicted

The best results were obtained for derivative **12**, which showed the highest probabilities for the mucomembranous protection, antieczematic, antiulcerative, and antiviral activity. The predictions for compounds **2**–**4** and **12** showed that they are also possible mucomembranous protectors. Moreover, derivatives **3**, **4** may be radioprotectors and may have antiviral activity. According to PASS calculations a possible mechanism of action for compounds **2**–**4** and **11**, **12** is enzymes inhibition: chloride peroxidase, muramoyltetrapeptide carboxypeptidase, Cl^−^-transporting ATPase. Other mechanisms of membrane permeability and gastrin inhibitions should be associated with mucomembranous protection and antiulcerative activity, respectively.

## Conclusion

In conclusion, we have successfully conducted simple synthesis leading to the compounds containing thiazole and 4,5-dihydrothiazole ring in the reaction of 1-allylthiourea with haloesters and oxoesters, respectively. By changing the reaction conditions we have managed to obtain the products of a very good performance. The method is very universal and can be applied for the synthesis of compounds containing thiazole and dihydrothiazole rings.

## Experimental

^1^H NMR and ^13^C NMR spectra were recorded on Bruker 300 MHz, 400 MHz, and 700 MHz apparatus (TMS as an internal standard). MS spectra were recorded on the Finnigan MAT 95. UV spectra were recorded on the spectrophotometer Aquarius 7250 Cecil Instruments. All chemicals and solvents were purchased commercially and used without further purification. The progress of reactions and also the purity of the obtained compounds were monitored by TLC on TLC-sheets ALUGRAM SIL G/UV_254_ plates with ethyl acetate as an eluent.

### General procedures for the synthesis of compounds 5–7, 11, 12

#### Procedure A

To a solution of sodium methoxide (prepared from 0.1 mol of sodium in 60 cm^3^ anhydrous MeOH), 0.05 mol of **1** and 0.055 mol of ester (**2–4**, **9**, **10**) were added and refluxed (reflux time depends on the compounds present in Tables 1, 2). The solvent was evaporated and the residue was dissolved in water and neutralized with HCl to pH 7–8. The product was extracted with CHCl_3_, dried, concentrated, and crystallized from diethyl ether.

#### Procedure B

To a solution of 30 cm^3^ anhydrous EtOH, 0.05 mol of **1** and 0.055 mol of ester (**2–4**, **9**, **10**) were added and refluxed (reflux time depends on the compounds present in Tables 1, 2). The solvent was evaporated and the residue was dissolved in water and neutralized with NaOH to pH 7–8. The product was extracted with CHCl_3_, dried, concentrated, and crystallized from diethyl ether.

#### Procedure C

To a solution of 30 cm^3^ anhydrous MeOH, 0.03 mol of triethyl orthoformate, 0.075 g *p*-toluenesulfonic acid and 0.05 mol of chlorooxoester **9** or **10** were added and mixed for 24 h. Diethyl ether (30 cm^3^) was added to the mixture and neutralized with sodium ethoxide to pH 7–8, then 30 cm^3^ distilled water was added. The product was extracted with diethyl ether and dried with sodium carbonate. The solvent was evaporated and to the residue 0.02 mol of **1**, and 0.11 cm^3^ of concentrated sulfuric acid were added and refluxed for 1 h. The solvent was evaporated and the residue was dissolved in 30 cm^3^ water and neutralized with NaOH to pH 7–8. The product was extracted with CHCl_3_, dried, concentrated, and crystallized from diethyl ether.

##### *2*-*(Allylamino)thiazol*-*4(5H)*-*one* (**5**, C_6_H_8_N_2_OS)

M.p.: 89–90 °C; UV (H_2_O + 5 % EtOH): *λ*_max_ (*ε* × 10^3^) = 191 (0.75), 230.5 (2.22) nm (mol^−1^ dm^3^ cm^−1^); ^1^H NMR (CDCl_3_): *δ* = 3.83 (s, 2H, CH_2_), 3.94 (t, *J* = 1.4 Hz, 1H, NH), 3.96 (dd, *J* = 1.4, 5.6 Hz, 2H, CH_2_=CH–C*H*_*2*_), 5.24 (dd, *J* = 2.1, 10.5 Hz, 1H, C*H*_*A*_H_B_=CH–CH_2_), 5.31 (dd, *J* = 2.1, 17.5 Hz, 1H, CH_A_*H*_*B*_=CH–CH_2_), 5.90 (m, 1H, CH_2_=C*H*–) ppm; ^13^C NMR (100 MHz, CDCl_3_): *δ* = 185.8, 183.8, 131.7, 118.6, 47.1, 38.7 ppm; MS (CI, isobutane): *m*/*z* (%) = 157 ([M + 1]^+^, 100).

##### *2*-*(Allylamino)*-*5*-*methylthiazol*-*4(5H)*-*one* (**6**, C_7_H_10_N_2_OS)

M.p.: 75–76 °C; UV (H_2_O + 5 % EtOH): *λ*_max_ (*ε* × 10^3^) = 192.5 (0.97), 231 (2.19) nm (mol^−1^ dm^3^ cm^−1^); ^1^H NMR (CDCl_3_): *δ* = 1.61 (d, *J* = 7.0 Hz, 2H, C–CH_3_), 3.91 (t, *J* = 1.4 Hz, 1H, NH), 3.93 (dd, *J* = 1.4, 5.6 Hz, 2H, CH_2_=CH–C*H*_*2*_), 4.08 (q, *J* = 7.0 Hz, C*H*–CH_3_), 5.23 (dd, *J* = 1.4, 10.5 Hz, 1H, C*H*_*A*_H_B_=CH–CH_2_), 5.31 (dd, *J* = 1.4, 17.5 Hz, 1H, CH_A_*H*_*B*_=CH–CH_2_), 5.90 (m, 1H, CH_2_=C*H*–) ppm; ^13^C NMR (100 MHz, CDCl_3_): *δ* = 189.0, 182.2, 131.7, 118.5, 49.6, 47.2, 19.0 ppm; MS (CI, isobutane): *m*/*z* (%) = 171 ([M + 1]^+^, 100).

##### *2*-*(Allylamino)*-*5*-*ethylthiazol*-*4(5H)*-*one* (**7**, C_8_H_12_N_2_OS)

M.p.: 77–78 °C; UV (H_2_O + 5 % EtOH): *λ*_max_ (*ε* × 10^3^) = 192 (0.85), 231.5 (2.31) nm (mol^−1^ dm^3^ cm^−1^); ^1^H NMR (CDCl_3_): *δ* = 0.99 (dt, *J* = 2.8, 7.0 Hz, 3H, CH–CH_2_–C*H*_*3*_), 2.00 (dq, *J* = 2.8, 4.2 Hz, 2H, CH–C*H*_*2*_–CH_3_), 3.95 (dd, *J* = 1.4, 5.6 Hz, 2H, CH_2_=CH–C*H*_*2*_), 4.09 (t, *J* = 1.4 Hz, 1H, NH), 4.18 (tq, *J* = 4.2, 7.0 Hz, C*H*–C_2_H_5_), 5.23 (dd, *J* = 1.4, 10.5 Hz, 1H, C*H*_*A*_H_B_=CH–CH_2_), 5.30 (dd, *J* = 1.4, 17.5 Hz, 1H, CH_A_*H*_*B*_=CH–CH_2_), 5.94 (m, 1H, CH_2_=C*H*–) ppm; ^13^C NMR (100 MHz, CDCl_3_): *δ* = 188.0, 182.6, 131.8, 118.4, 57.4, 47.3, 26.2, 11.5 ppm; MS (CI, isobutane): *m*/*z* (%) = 185 ([M + 1]^+^, 100).

##### *Ethyl 2*-*(allylamino)*-*4*-*methylthiazole*-*5*-*carboxylate* (**11**, C_10_H_14_N_2_O_2_S)

M.p.: 104–105 °C; UV (H_2_O + 5 % EtOH): *λ*_max_ (*ε* × 10^3^) = 216 (9.77), 308 (17.73) nm (mol^−1^ dm^3^ cm^−1^); ^1^H NMR (CDCl_3_): *δ* = 1.32 (t, *J* = 6.9 Hz, 3H, OCH_2_C*H*_*3*_), 1.87 (1H, NH), 2.51 (s, 3H, C^4^–C*H*_*3*_), 3.88 (d, *J* = 5.2 Hz, 2H, CH_2_=CH–C*H*_*2*_), 4.25 (q, *J* = 7.1 Hz, 2H, OC*H*_*2*_CH_3_), 5.23 (dd, 1H, *J* = 1.5, 10.2 Hz, C*H*_*A*_H_B_=CH–CH_2_), 5.33 (dd, *J*=1.5, 17.3 Hz, 1H, CH_A_*H*_*B*_=CH–CH_2_), 5.88 (m, 1H, CH_2_=C*H*–) ppm; ^13^C NMR (100 MHz, CDCl_3_): *δ* = 171.6, 162.7, 159.4, 132.5, 117.7, 109.3, 60.4, 48.3, 17.4, 14.5 ppm; MS (CI, isobutane): *m*/*z* (%) = 227 ([M + 1]^+^, 100).

##### *Ethyl 2*-*[2*-*(allylamino)thiazol*-*4*-*yl]acetate* (**12**, C_10_H_14_N_2_O_2_S)

M.p.: 42–43 °C; UV (H_2_O + 5 % EtOH): *λ*_max_ (*ε* × 10^3^) = 261 (7.55) nm (mol^−1^ dm^3^ cm^−1^); ^1^H NMR (CDCl_3_): *δ* = 1.28 (t, *J* = 6.9 Hz, 3H, OCH_2_C*H*_*3*_), 1.93 (1H, NH), 3.57 (d, *J* = 0.9 Hz, 2H, C*H*_*2*_OCH_2_CH_3_), 3.86 (d, *J* = 5.2 Hz, 2H, CH_2_=CH–C*H*_*2*_), 4.21 (q, *J* = 6.9 Hz, 2H, OC*H*_*2*_CH_3_), 5.20 (dd, *J* = 1.5, 11.1 Hz, 1H, C*H*_*A*_H_B_=CH–CH_2_), 5.31 (dd, *J*=1.5, 17.4 Hz, 1H, CH_A_*H*_*B*_=CH–CH_2_), 5.91 (m, 1H, CH_2_=C*H*–), 6.34 (t, *J* = 0.9 Hz, 1H, C^5^H) ppm; ^13^C NMR (100 MHz, CDCl_3_): *δ* = 170.5, 169.7, 145.0, 133.5, 117.2, 103.6, 60.9, 48.3, 37.4, 14.2 ppm; MS (70 eV): *m*/*z* = 226 ([M + 1]^+^, 100).

### X-ray diffraction study

The X-ray data for reported structures were collected at 293(2) K with an Oxford Sapphire CCD diffractometer using MoKα radiation *λ* = 0.71073 A and *ω*-2*θ* method. The numerical absorption corrections were applied (RED171 package of programs Oxford Diffraction, 2000) [22]. All structures have been solved by direct methods and refined with the full-matrix least-squares method on *F*^2^ with the use of SHELX-97 program package [23]. The hydrogen atoms have been located from the different electron density maps and constrained during refinement. The crystallographic data have been deposited with the Cambridge Crystallographic Data Centre, the CCDC numbers: 1034864 for **5** and 1034865–1034867 for **7**, **11**, and **12**, respectively.

## References

[1] Barf T, Vallgårda J, Edmont R, Häggström C, Kurz G, Nygren A, Larwood V, Mosialou E, Axelsson K, Olsson R, Engblom L, Edling N, Rönquist-Nii Y, Öhman B, Alberts P, Abrahmsén L (2002). J Med Chem.

[2] Fotsch C, Wang M (2008). J Med Chem.

[3] Hale C, Véniant M, Wang Z, Chen M, McCormick J, Cupples R, Hickman D, Min X, Sudom A, Xu H, Matsumoto G, Fotsch C, St Jean DJ, Wang M (2008) Chem Biol Drug Des 71:36 10.1111/j.1747-0285.2007.00603.x 18069989

[4] Jean DJ, Yuan C, Bercot EA, Cupples R, Chen M, Fretland J, Hale C, Hungate RW, Komorowski R, Veniant M, Wang M, Zhang X, Fotsch C (2007). J Med Chem.

[5] Johansson L, Fotsch C, Bartberger DM, Castro VM, Chen M, Emery M, Gustafsson S, Hale C, Hickman D, Homan E, Jordan SR, Komorowski R, Li A, McRae K, Moniz G, Matsumoto G, Orihuela C, Palm G, Veniant M, Wang M, Williams M, Zhang J (2008). J Med Chem.

[6] Yuan C, St Jean DJ Jr, Liu Q, Cai L, Li A, Han N, Moniz G, Askew B, Hungate RW, Johansson L, Tedenborg L, Pyring D, Williams M, Hale C, Chen M, Cupples R, Zhang J, Jordan S, Bartberger MD, Sun Y, Emery M, Wang M, Fotsch C (2007) Bioorg Med Chem Lett 17:6056 10.1016/j.bmcl.2007.09.070 17919905

[7] Marsham PR, Hughes LR, Jackman AL, Hayter AJ, Oldfield J, Wardleworth JM, Bishop JA, O’Connor BM, Calvert AH (1991). J Med Chem.

[8] Srivastava W, Gupta SP, Siddiqi MI, Mishra BN (2010). Eur J Med Chem.

[9] Levshin IB, Grigor’eva IV, Tsurkan AA, V’yunov KA, Ginak AI (1985) Chem Heterocycl Comp 21:277

[10] Schubert H, Scholz J (1955) Verfahren zur Herstellung von substituierten Pseudothiohydantoinhydrohalogeniden. Patent DE 936688, Dec 22, 1955; (1959) Chem Abstr 53:34867

[11] Studzińska R, Wróblewski M, Dramiński M (2008). Heterocycles.

[12] Studzińska R, Wróblewski M, Karczmarska-Wódzka A, Kołodziejska R (2014). Tetrahedron Lett.

[13] Turski K, Dramiński M (1992). Pol J Chem.

[14] Turski K, Dramiński M (1993). Pol J Chem.

[15] Zav’yalov SI, Dorofeeva OV, Rumyantseva EE, Kulikova LB, Ezhova GI, Kravchenko NE, Zavozin AG (2001) Pharm Chem J 35:96

[16] Gagiu F, Draghici C, Banu E, Csavassy Gh, Vrejoiu G, Theodorescu M (1970). Chim Ther.

[17] Lipinski CA, Lombardo F, Dominy BW, Feeney PJ (2012). Adv Drug Deliver Rev.

[18] Molinspiration Cheminformatics. www.molinspiration.com (Accessed February 16, 2015)

[19] Ertl P, Rohde B, Selzer P (2000). J Med Chem.

[20] PASS on line. http://www.pharmaexpert.ru/passonline. (Accessed Feb 16, 2015)

[21] Filimonov DA, Lagunin AA, Gloriozova TA, Rudik AV, Druzhilovskii DS, Pogodin PV, Poroikov VV (2014). Chem Heterocycl Comp.

[22] CrysAlis CCD171 and RED171 package of programs (2000) Oxford Diffraction

[23] Sheldrick GM (2008). Acta Cryst A.

